# NVTrans‐UNet: Neighborhood vision transformer based U‐Net for multi‐modal cardiac MR image segmentation

**DOI:** 10.1002/acm2.13908

**Published:** 2023-01-18

**Authors:** Bingjie Li, Tiejun Yang, Xiang Zhao

**Affiliations:** ^1^ School of Information Science and Engineering Henan University of Technology Zhengzhou China; ^2^ School of Artificial Intelligence and Big Data Henan University of Technology Zhengzhou China; ^3^ Key Laboratory of Grain Information Processing and Control (HAUT) Ministry of Education Zhengzhou China; ^4^ Henan Key Laboratory of Grain Photoelectric Detection and Control (HAUT) Zhengzhou Henan China

**Keywords:** cardiac pathology segmentation, fuzzy boundary, multi‐modal fusion, transformer, U‐Net

## Abstract

With the rapid development of artificial intelligence and image processing technology, medical imaging technology has turned into a critical tool for clinical diagnosis and disease treatment. The extraction and segmentation of the regions of interest in cardiac images are crucial to the diagnosis of cardiovascular diseases. Due to the erratically diastolic and systolic cardiac, the boundaries of Magnetic Resonance (MR) images are quite fuzzy. Moreover, it is hard to provide complete information using a single modality due to the complex structure of the cardiac image. Furthermore, conventional CNN‐based segmentation methods are weak in feature extraction. To overcome these challenges, we propose a multi‐modal method for cardiac image segmentation, called NVTrans‐UNet. Firstly, we employ the Neighborhood Vision Transformer (NVT) module, which takes advantage of Neighborhood Attention (NA) and inductive biases. It can better extract the local information of the cardiac image as well as reduce the computational cost. Secondly, we introduce a Multi‐modal Gated Fusion (MGF) network, which can automatically adjust the contributions of different modal feature maps and make full use of multi‐modal information. Thirdly, the bottleneck layer with Atrous Spatial Pyramid Pooling (ASPP) is proposed to expand the feature receptive field. Finally, the mixed loss is added to the cardiac image to focus the fuzzy boundary and realize accurate segmentation. We evaluated our model on MyoPS 2020 dataset. The Dice score of myocardial infarction (MI) was 0.642 ± 0.171, and the Dice score of myocardial infarction + edema (MI + ME) was 0.574 ± 0.110. Compared with the baseline, the MI increases by 11.2%, and the MI + ME increases by 12.5%. The results show the effectiveness of the proposed NVTrans‐UNet in the segmentation of MI and ME.

## 1 INTRODUCTION

The cardiac is one of the most important organs in the human body, which is responsible for blood circulation. The prevalence and mortality of cardiovascular disease are still on the rise. Among non‐communicable illnesses, cardiovascular disease continues to be the major cause of mortality, the mortality rate ranks first.^1^ By 2030, it is anticipated that there will be more than 23 million cardiovascular disease‐related deaths worldwide.^2^ In addition, acute myocardial infarction is the major cause of mortality from cardiovascular disease and has become the main disease risk.^3^ In clinical practice, accurate segmentation of cardiac substructure and diseased tissue is the essential premise of cardiovascular disease (CVD) diagnosis, prevention, and assisting doctors in treatment.^4^, ^5^ Cardiac images are generally manually segmented by doctors or experts based on existing medical knowledge, clinical experience, and medical conditions, but this method is time‐consuming, energy‐consuming, and highly subjective.^6^ At the same time, they can also lead to inaccurate segmentation in the case of long‐term fatigue work. Therefore, for the precise diagnosis and prompt treatment of cardiovascular diseases, automated segmentation is crucial.

Despite many methods that have been applied for cardiac segmentation, the cardiac is a dynamic organ and its shape will change during the beating process, which is prone to noise and artifacts. The difficulty of localization and segmentation increases. In cardiac image segmentation, the shape and working mode of each region is different, which makes the segmentation algorithm of pathological regions difficult to achieve the desired effect. In addition, due to the difficulty in extracting complex features of cardiac structure, the fuzzy boundary of pathological regions in the cardiac image and the limitation of unimodal information, the segmentation task still has a great challenge. In summary, our primary aim is to analyze how to achieve accurate and efficient segmentation of pathological regions (MI and ME) in cardiac images.

Over the past few years, several CMR image segmentation techniques have been proposed. We usually divide the advanced methods into the unimodal method and multi‐modal method in recent years.

Firstly, some advanced methods have recently been developed for unimodal segmentation of cardiac images. For example, in 2021, Bi et al.^7^ proposed a model to extract the left ventricle and discussed a sequential shape similarity (SSS), which is based on the Active Contour Model. The base model can accurately outline the boundary of the left ventricle with the snake contour algorithm under the constraint of SSS. Ammar et al.^8^ evaluated a U‐Net‐based variant, a 2D convolution long short‐term memory (LSTM) recurrent neural network, by capturing the potential relevance. Cui et al.^9^ developed an attention U‐Net architecture, which pays more attention to the region of interest (ROI) with distinct sizes and shapes automatically, highlights the required parts while suppressing irrelevant regions. It effectively solves the problem of a high imbalance between the ROI and background regions. The accuracy of cardiac image segmentation is effectively improved. Nevertheless, in clinical practice, the limitations of unimodal obstruct the practical use of computer‐assisted diagnostics.

Due to the limitations of unimodal, multi‐modal segmentation has attracted more and more attention. Doctors can more accurately localize and diagnose lesion regions with the help of multi‐modal information. Therefore, amount of multi‐modal segmentation methods have been introduced recently, and the segmentation results show the superiority of multi‐modal compared with unimodal. For example, in 2019, Zhou et al.^10^ presented a review of multi‐modal segmentation methods and analyzed the fusion strategies of different network structures. In 2020, Zhang et al.^11^ introduced a multi‐modal cardiac pathology segmentation architecture using a fusion algorithm. The architecture is mainly composed of two neural networks: anatomical structure segmentation network (ASSN) and pathological regions segmentation network (PRSN). Liao et al.^12^ designed a multi‐modal transfer learning network based on adversarial training for 3D cardiac segmentation. The spatial attention mechanism is introduced to optimize feature extraction and remove redundant information. To address the difficult segmentation caused by the diversity of lesion regions. In 2022, Li et al.^13^ evaluated a Siamese U‐Net, which first explored the correlation among multi‐modal, and secondly extracted ROI features to improve the fusion of information. In summary, there are several applications for the multi‐modal image in the diagnosis of cardiac diseases. Due to the uneven grayscale of MRI images and different imaging methods showing different tumor substructures, it is necessary to set multi‐modal segmentation tasks by using the characteristics of multi‐modal MRI images.

The misalignment of multi‐modal due to various scanning directions, and the low tissue contrast of special modalities make multi‐modal segmentation tasks great challenges.^14^ This paper mainly studies the multi‐modal cardiac segmentation, in which the automatic segmentation of MI and MI + ME is the focus of the research. Herein, we design NVTrans‐UNet for multi‐modal cardiac image segmentation. Three parallel encoders are used to extract features from three modalities,^15^ to adapt to the difference in the pixel intensity distribution of each modality. The summary of our major contributions is as follows:

1. In the encoding phase, we leverage an efficient hierarchical neighborhood Transformer, namely NVT. NVT utilizes overlapping small convolution kernels for feature embedding and down sampling, which pays more attention to local information and has low complexity.
2. We introduce the MGF network to each Transformer layer of the encoder. The network aggregates the feature maps of task‐related information in the three modalities and automatically learns to adjust the contribution of the three modal feature maps.
3. A bottleneck layer with ASPP is added between the encoder and the decoder, which can accurately capture information of different scales and increase the capacity to express detailed features, as well as the ability to recognize and segment small objects.
4. The mixed loss is introduced to optimize the network, allowing the model to focus on the boundaries of myocardial pathology while simultaneously resolving the issue of class imbalance.

The following is how this article is organized. We introduce the network architecture for cardiac segmentation in section 2, and the related work of different modules under the overall framework. Section 3 describes the dataset, experimental configuration, and evaluation metric. Section 4 describes the experimental results and ablation experiments. The fifth section is the discussion and prospect, and the sixth section is the summary.

## 2 METHOD

We introduce the presented NVTrans‐UNet network and describe thoroughly the overall design of the network, Neighborhood Vision Transformer (NVT), multi‐modal gated fusion module (MGF), Atrous Spatial Pyramid Pooling (ASPP), and loss function.

### 2.1 Network architecture

**FIGURE 1:**
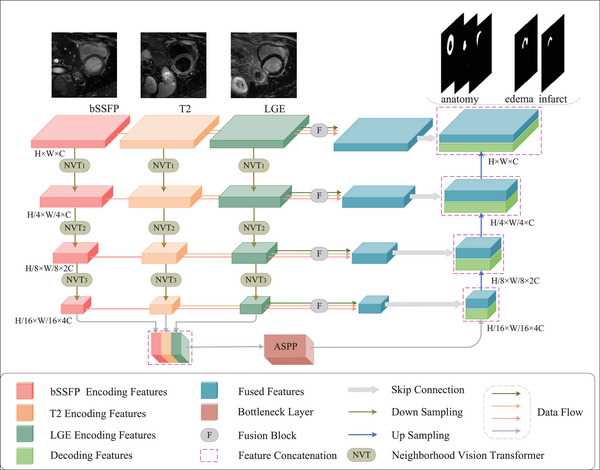
The overview architecture of the NVTrans‐UNet segmentation model.

The NVTrans‐UNet architecture is mainly made up of the following modules: encoder module, bottleneck layer, and decoder module. In general, we introduce NVT and MGF to fully utilize the local and multi‐modal information respectively. ASPP is added to the bottleneck layer to expand the receptive field, decrease the number of parameters and enhance the capacity to extract detailed features. Figure 1 demonstrates the overview architecture of our model.

### 2.2 Neighborhood vision transformer

**FIGURE 2:**
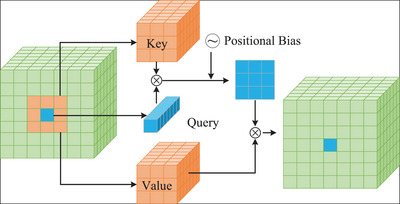
Multi‐headed neighborhood attention block.

Since self‐attention (SA) cannot handle long sequences in Transformer architecture, it needs large memory and high time complexity for high‐resolution images. Therefore, we leverage NVT, which is built on a simple and flexible attention mechanism NA, which localizes the receptive field of each token to its nearest neighboring pixel. The multi‐headed neighborhood attention block is shown in Figure 2. NA is a localization of SA, in which complexity is linear for the resolution and also for the neighborhood size. Compared to SA, NA not only reduces the computational cost but also includes local inductive biases similar to the convolution operation.

**FIGURE 3:**
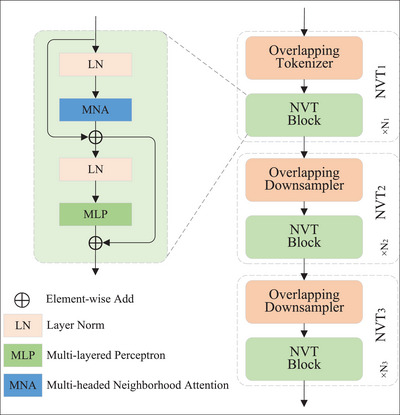
Neighborhood vision transformer structure.

The model starts with a convolutional downsampler and then consists of three sequential layers, each consisting of multiple NVT blocks. The first layer of NVT downsamples the input feature using two consecutive 3 × 3 convolutions with 2 × 2 strides to make the spatial size $\frac{1}{4}$ of the input size. Each NVT block consists of multi‐headed neighborhood attention (NA), a multi‐layered perceptron (MLP), layer norm (LN), and skip connections. Figure 3 depicts the structure of NVT. The downsampler doubles the number of channels while halving the spatial size. This simplifies the computation of subsequent levels. Our model generates feature maps of size $\frac{H}{4}\times \frac{W}{4}$, $\frac{H}{8}\times \frac{W}{8}$, and $\frac{H}{16}\times \frac{W}{16}$. It is computed as follows:

(1) $$MNA\left(X_{i,j}\right)=softmax\left(\frac{Q_{i,j}K_{\rho (i,j)}^{T}+B_{i,j}}{scale}\right)V_{\rho (i,j)}$$

where ρ(i,j) denotes the neighborhood of a pixel at (i,j). $|\left|\rho (i,j)\right||\;=S^{2}\;$ (*S* is the neighborhood size), *Q*, *K*, and *V* are linear projections of input *X*, $B_{i,\;j}$ is the relative position bias.

### 2.3 Multi‐modal gated fusion

Because different modalities reflect diverse sub‐structures of the cardiac, their contribution weights in various cardiac sub‐structures differ. But the existing classical fusion strategies have the problem that the contributions of different modalities cannot be dynamically balanced.^16^ Consequently, we introduce a multi‐modal gated fusion (MGF) strategy, which can automatically learn to adjust the contribution of feature mapping from each modal. At this point, we learn a weighted mapping dynamically to control the proportion from each modal information. Then, the features of each modal are fused. To realize the multi‐scale fusion of multi‐modal information, we perform the MGF module in each NVT layer in the encoder.

**FIGURE 4:**
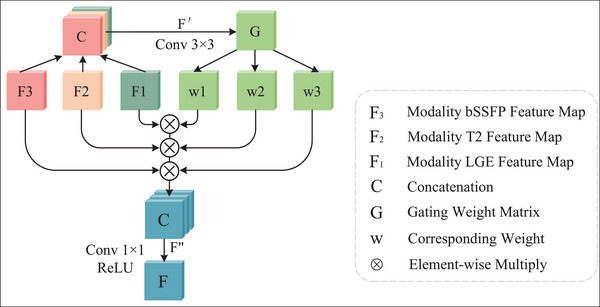
Structure of the multi‐modal gated fusion (MGF).

The MGF is superior to the existing conventional fusion strategies by properly aggregating the complementary information with correlation weight. Figure 4 depicts the MGF module structure. Specifically, the MGF concatenates the features from each downsampling layer and inputs them to the upsampling layers with three output channels. Then the weight matrix G is generated by the sigmoid activation function. This matrix can be divided into three independent maps of {*W*_1_, *W*_2_, *W*_3_}, which are multiplied by the feature maps of three modalities, and then the outputs are concatenated. Each weight is a trainable parameter. During the training phase, the optimizer continuously updates the parameters to minimize the loss function. Eventually, we get the fusion result *F*. The output has the same feature mapping size and channel number as the input. Mathematically, the MGF can be expressed as:

(2) $$\begin{array}{ccc} F^{{}^{\prime }} & = & F_{1}⊕F_{2} \end{array}⊕F_{3}$$

(3) $$W_{i}=\sigma \left(C_{i}*F^{{}^{\prime }}+b_{i}\right),i=1,2,3$$

(4) $$\begin{array}{c} F^{{}^{\prime \prime }}\left(i\right)=\left(F_{1}\left(i\right)⊗W_{1}\right)⊕\left(F_{2}\left(i\right)⊗W_{2}\right)⊕\left(F_{3}\left(i\right)⊗W_{3}\right) \end{array}$$

(5) $$F=ReLU(C_{j}*F^{{}^{\prime \prime }}+b_{j})$$

where σ is the sigmoid function, and σ(x)Δ=11+e−x, ⊕ represents the concatenation, $C_{i}$ and $C_{j}$ are the convolution kernel size, ⊗ denotes the element‐wise product, $F^{\prime \prime }\left(i\right)$ refers to ith feature map of *F*, $b_{i}$, and $b_{j}$ are biases of the convolution layers.

### 2.4 Atrous spatial pyramid pooling

**FIGURE 5:**
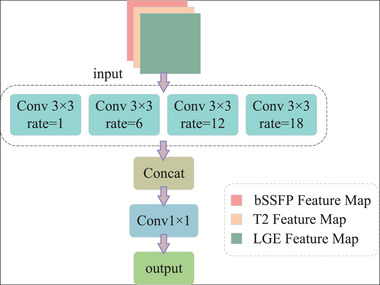
Structure of the Atrous Spatial Pyramid Pooling (ASPP).

The bottleneck layer provides a connection between the encoder and decoder, offering multi‐scale information for semantic segmentation tasks. Therefore, we add ASPP to the bottleneck layer to increase the receptive field and robustly segment object regions at different levels.^17^ The structure of ASPP is shown in Figure 5.

In order to extract contextual information in the multi‐scale features and recover spatial information from diverse field‐of‐views, ASPP employs filters with multiple sampling rates and effective field‐of‐view to detect incoming convolutional feature layers. Four parallel distributed atrous convolutions (*r* = {1, 6, 12, 18}) are used to combine features from distinct receptive fields. The features extracted from each sampling layer are further processed in various branches and concated to produce the final result. As well as increasing the feature receptive field, ASPP can also improve the recognition and detection of small objects. The ASPP can be stated mathematically as:

(6) $$O\;=\frac{i+2p-k-\left(k-1\right)\left(d-1\right)}{s}\;+1$$

where *i* represents the kernel size of input atrous convolution, *p* denotes the size of the padding, and *s* indicates stride size. *k* is the size of the original convolution kernel, *d* stands for dilation‐rate, k+(k−1)(d−1) is the convolution kernel size after the dilation‐rate is inserted. O refers to the size of the feature map after atrous convolution.

### 2.5 Loss function

Since the pathological regions in the cardiac image are relatively small, the segmentation of the cardiac image is unbalanced. In unbalanced segmentation, there may be large differences among different regions, and the boundary of the pathological regions of the cardiac image is fuzzy, which will affect the training effect.^18^

We create a multi‐scale structural similarity index measure (MS‐SSIM)^19^ loss function to apply larger weights to the boundary in order to further improve the boundary of pathological regions. The larger MS‐SSIM value indicates a larger regional distribution difference. The segmentation result *p* and the ground truth label *t* are cropped into two identical N×N sized patches, which have been aligned with each other. Let $p\;=\;\{p_{i}|i=1,\;2,\text{…},N^{2}\}$ and $t=\{t_{i}|i=1,\;2,\text{…},N^{2}\}$, the luminance, contrast, structure comparison measures and MS‐SSIM loss function are given as follows:

(7) $$l_{M}\left(p,t\right)\;=\frac{2\mu_{p}\mu_{t}+C_{1}}{\mu_{p}^{2}+\mu_{t}^{2}+C_{1}}\;$$

(8) $$c_{m}\left(p,t\right)\;=\frac{2\sigma_{p}\sigma_{t}+C_{2}}{\sigma_{p}^{2}+\sigma_{t}^{2}+C_{2}}\;$$

(9) $$s_{m}\left(p,t\right)\;=\frac{\sigma_{pt}+C_{3}}{\sigma_{p}\sigma_{t}+C_{3}}\;$$

(10) $$\begin{array}{c} l_{MS-SSIM\left(p,t\right)}=\;1-{\left[l_{M}\left(p,t\right)\right]}^{\alpha_{M}}\prod_{m\;=\;1}^{M}{\left[c_{m}\left(p,t\right)\right]}^{\beta_{m}}{\left[s_{m}\left(p,t\right)\right]}^{\gamma_{m}} \end{array}$$

where μ_p_, μ_t_ are the mean and σ_p_, σ_t_ are the standard deviations of the predicted image and the ground truth respectively. $\sigma_{pt}$ represents the covariance between the predicted image and the ground truth. *C*_1_, $C_{2\;}$, and *C*_3_ are constants. *M* denotes the total number of scales. $\alpha_{M}$, $\beta_{m}$, and $\gamma_{m}$ are employed to modify the three elements' respective relative weights.

By combining MS‐SSIM loss,^19^ Tversky loss,^20^ and Focal loss,^21^ we design a mixed loss function for segmentation in patch level and pixel level hierarchies, which can capture fine structures with clear boundaries.

In order to reduce the weight of massive volumes of negative data in training, the Focal loss function is primarily employed to address the issue of class imbalance between positive and negative samples.^22^ The following is the Focal loss formula:

(11) $$\;l_{F}=\sum_{H,\;W}\left[-Y_{t}{\left(1-Y_{p}\right)}^{\gamma }log\left(Y_{p}\right)\right]\;$$

*Y*_p_ represents the predicted segmentation result and *Y*_t_ represents the ground‐truth. ${\left(1-Y_{p}\right)}^{\gamma }\;$represents the modulating factor. γ indicates the focusing parameter. The impact of the modulation factor similarly grows as γ increases. The purpose of adding this modulation factor is to reduce the weight of easy‐to‐classify pixels so that the model can focus more on hard‐to‐classify pixels during training.

In the case of sample imbalance, misclassification can lead to a large increase in loss, leading to unstable optimization. Abraham et al.^23^ studied the similarity index based on Tversky, which introduced two parameters (α and β) to control false positives (FP) and false negatives (FN), the balance between FP and FN can be adjusted by α and β. The Tversky loss formula is:

(12) $$\begin{array}{c} \;l_{T}=\;1-\frac{1+Y_{t}Y_{p}}{1+Y_{t}Y_{p}+\alpha \left(1-Y_{t}\right)Y_{p}+\beta Y_{t}\left(1-Y_{p}\right)} \end{array}$$

*Y*_t_ and *Y*_p_ refer to the real label and the prediction result respectively. The sum of α and β is 1. Consequently, the mixed loss function is as follows:

(13) $$L\;=\;\gamma l_{MS-SSIM}+\eta l_{F}+\theta l_{T}$$

Where the γ, η, and θ are trade‐off parameters weighting the impact of each term, γ is empirically set as 5 and η=3θ in our experiments.

## 3 EXPERIMENTS

### 3.1 Dataset and preprocessing

We test our model using the MyoPS 2020 dataset for myocardial pathology segmentation, which contains three modalities, including Late Gadolinium enhancement (LGE) CMR, T2‐weighted CMR, and balanced steady‐state free precession (bSSFP) CMR. LGE CMR is bright in the region of MI, it can be used to identify regions of inflammation, cardiomyopathy and infarct^24^ but its anatomical and edema boundaries are fuzzy. The bSSFP CMR can capture cardiac motion and the boundary between the myocardium and blood cavity.^25^ T2‐weighted is helpful to distinguish acute myocardial infarction from distal myocardial infarction. It could simultaneously segment MI and ME by combining the three modalities. MyoPS 2020 contains 25 labeled (102 slices) multi‐sequence CMR images and 20 unlabeled (72 slices) images. We first slice the cardiac volumes into 2D images. Considering that the ROI only occupies a small part of the whole image, then crop the center to 288 × 288 pixels. Given the small number of samples, we implement a data argumentation strategy by random warping and rotation. Firstly, the random warping is achieved by generating an 8 × 8 × 2 evenly distributed random matrix. Then, we adjusted the size of the nonrigid warping matrix to 288 × 288 × 2, and used the bi‐linear interpolation method to process the warping map. After using random warping to augment the data, we selected with equal probability at 90°, 180°, and 270° using random rotation.

**FIGURE 6:**
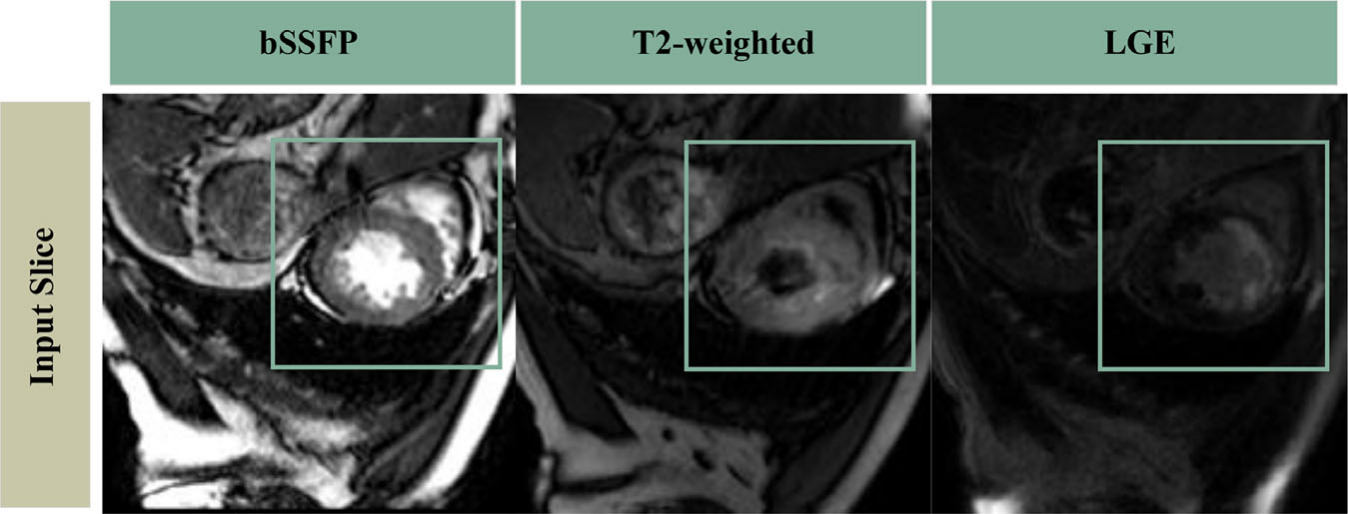
The input multi‐modal cardiac image slice.

**FIGURE 7:**
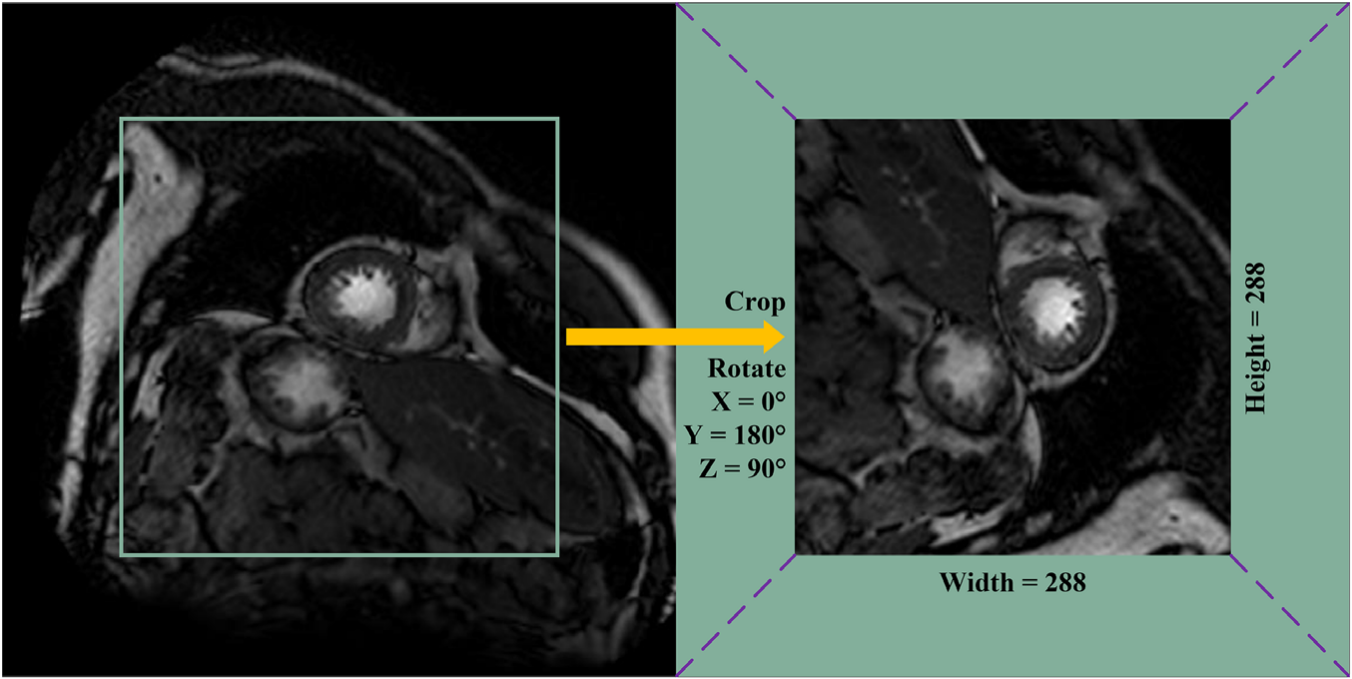
Schematic diagram of center region preprocessing.

Figure 6 shows the input multi‐modal cardiac image slice and Figure 7 shows the schematic diagram of preprocessing approach. For all cardiac images, there are two pathological masks (myocardial infarction and edema) and three anatomy masks (myocardium, left ventricle, and right ventricle).

### 3.2 Experimental settings

The NVTrans‐UNet is implemented by Python based on TensorFlow (version 1.8.0). The hardware configuration uses a GeForce RTX 2080 Ti GPU with 11G memory. On the MyoPS 2020 public dataset, the training set and testing set are divided in a ratio of 4:1 and a 5‐fold cross‐validation was performed on the dataset. Batch normalization and ReLU activation functions are utilized to avoid vanishing gradients. During the training, we use the Adam optimizer with a learning rate of $1e^{-4}$ to optimize the network. We set 200 epochs of training and the batch size is 4.

### 3.3 Evaluation metric

The evaluation metrics used are Dice Similarity Coefficient (DSC) and Hausdorff Distance (HD) in this experiment. DSC and HD are often used to evaluate the quality of medical image segmentation. DSC measures the similarity between the segmentation results and ground truth. The specific formulation of DSC is as follows:

(14) $$Dice\left(P,T\right)\;=\frac{P\cap T}{\left(P+T\right)/2}\;$$

In Equation (14), *T* is the label, andPis the segmentation result of the evaluation method. The value range of Dice score is between 0 and 1.

HD is a measure that describes the degree of similarity between two point sets. The HD can be formulated as:

(15) $$\begin{array}{c} HD\left(P,\;T\right)\;=\;max\left\{{}_{S_{P}\in S\left(P\right)}^{max\;}d\left(S_{P},S\left(T\right)\right),\;{}_{S_{T}\in S\left(T\right)}^{max\;}d\left(S_{T},S\left(P\right)\right)\right\} \end{array}$$

In Equation (15), where Tdenotes labels and *P* represents predicted results, *S*_P_ and *S*_T_ are elements in two sets respectively. *d* express for the Euclidean distance.

## 4 RESULTS

### 4.1 Experiment results

To verify the effectiveness of the employed NVTrans‐UNet in multi‐modal cardiac image segmentation, we compared it with other advanced methods. These methods include baseline MFU‐Net,^26^ FCDensenet,^27^ FADLS,^28^ U‐Net,^29^ PyMIC,^30^ MVMM,^14^ and CMRadjustNet.^31^ The results show that the Dice score of MI was 0.642 ± 0.171, and the Dice score of MI + ME was 0.574 ± 0.110. Table 1 demonstrates the comparison of segmentation results with different methods.

**TABLE 1:** The comparison of results with other methods

**Methods**	**MI+ME**		**MI**	
	**dice**	**std**.	**dice**	**std**.
MFU‐Net^26^	0.449	0.139	0.530	0.205
FCDensenet^27^	0.540	0.229	0.579	**0.148**
FADLS^28^	0.557	0.183	0.468	0.268
U‐Net^29^	0.510	–	0.607	–
PyMIC^30^	0.525	–	0.637	–
MVMM^14^	**–**	**–**	0.524	0.158
CMRadjustNet^31^	0.542	0.241	0.628	0.277
Ours	**0.574**	**0.110**	**0.642**	0.171

Abbreviation: std, standard deviation.

Compared with FCDensenet, the Dice score of our model increased by 3.4% and 6.3% in MI + ME and MI, respectively. Compared with CMRadjustNet, the Dice score of our model increased by 3.2% and 1.4% in MI + ME and MI, respectively. Table 2 demonstrates that the segmentation results of our method in the cardiac pathological regions are significantly better than other methods. The MGF module in NVTrans‐UNet can take full advantage of the complementarity among multi‐modal information. The NVT module can extract the local information of the cardiac image. The ASPP can obtain more effective receptive fields and reduce the parameter size of the model. In addition, the boundary loss can enhance the boundary of the cardiac image, and give higher weights to the fuzzy boundary. All these show that the proposed NVTrans‐UNet has better segmentation performance.

**TABLE 2:** Comparison of dice scores with a different variant of our model

**Models**	**MGF**	**NVT**	**ASPP**	**ML**	**MI+ME**		**MI**		**Myo**.		**LV**		**RV**	
					**dice**	**std**.	**dice**	**std**.	**dice**	**std**.	**dice**	**std**.	**dice**	**std**.
M‐0					0.449	0.139	0.530	0.205	0.843	0.079	0.875	0.071	0.785	0.142
M‐1	√				0.465	0.195	0.559	0.215	0.848	0.055	0.881	0.031	0.812	0.078
M‐2		√			0.473	0.186	0.541	0.227	0.852	0.070	0.897	0.027	0.773	0.116
M‐3			√		0.486	0.161	0.584	0.196	0.820	0.093	0.880	0.026	0.795	0.122
M‐4				√	0.480	0.169	0.579	0.213	0.851	0.048	0.884	0.022	0.794	0.121
M‐5	√	√			0.488	0.132	0.590	0.190	0.848	0.071	0.885	0.028	0.789	0.130
M‐6	√		√		0.490	0.156	0.594	0.173	0.848	0.080	0.891	0.037	0.826	0.107
M‐7	√			√	0.498	0.171	0.619	0.215	0.857	0.086	0.883	**0.018**	0.803	0.128
M‐8		√	√		0.506	0.179	0.601	0.181	0.860	0.068	0.901	0.025	0.820	0.124
M‐9			√	√	0.489	**0.109**	0.590	0.142	0.844	0.051	0.884	0.051	0.826	0.120
M‐10		√		√	0.493	0.110	0.591	0.162	0.843	0.109	0.902	0.021	0.802	0.083
M‐11	√		√	√	0.515	0.125	0.626	0.110	0.861	0.070	0.906	**0.018**	0.817	0.122
M‐12	√	√		√	0.554	0.135	0.631	0.161	0.856	0.022	0.909	0.033	0.818	0.121
M‐13		√	√	√	0.543	0.164	0.639	**0.105**	0.846	0.030	0.913	0.030	0.796	0.112
M‐14	√	√	√		0.537	0.111	**0.642**	0.142	0.849	0.035	0.905	0.023	0.828	0.082
M‐15	√	√	√	√	**0.574**	0.110	**0.642**	0.171	**0.868**	**0.015**	**0.914**	0.029	**0.848**	**0.106**

### 4.2 Ablation study

**FIGURE 8:**
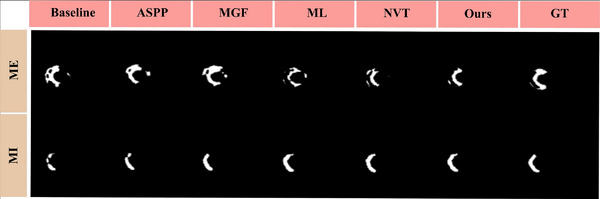
Visualization comparison of pathological regions segmentation effects of multi‐modal cardiac images under different ablation studies. The segmented regions include MI and ME. Where ASPP represents Atrous Spatial Pyramid Pooling, MGF represents multi‐modal gated fusion, ML represents mixed loss function, NVT represents Neighborhood Vision Transformer, and GT represents ground truth, respectively.

**FIGURE 9:**
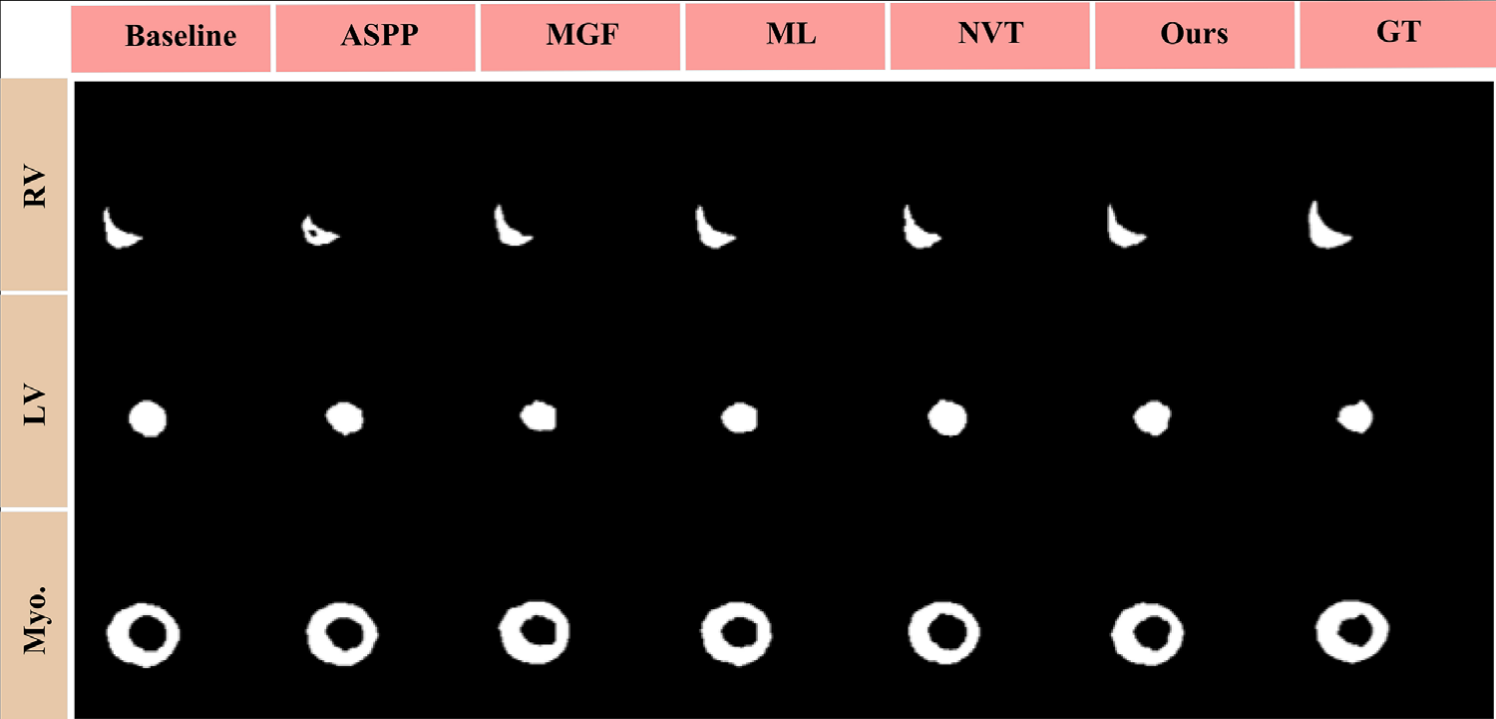
Visualization comparison of anatomical regions segmentation effects of multi‐modal cardiac images under different ablation studies. The segmented regions include Myo., LV, and RV.

To evaluate the impact of each component, we conducted detailed ablation experiments to quantify individual performance of different modules we introduced. Tables 2 and  3 exhibit the performance comparisons of different variants of our model under Dice scores and HD, respectively. In addition, the M‐0 to M‐15 represent different models that add the MGF, NVT, ASPP, and ML respectively, in which M‐0 denotes baseline and M‐15 denotes our model. Compared with baseline, our method increased by 11.2% of MI and 12.5% of MI + ME in Dice coefficient. Figures 8 and 9 show the comparison of pathological and anatomical qualitative results of different variants with baseline, respectively. It demonstrates that the model we proposed has better performance in segmenting small‐size regions MI and ME.

**TABLE 3:** Performance comparison of Hausdorff distance with multiple strategies on the test set

**Models**	**MGF**	**NVT**	**ASPP**	**ML**	**Hausdorff distance(mm)**				
					**MI+ME**	**MI**	**Myo**.	**LV**	**RV**
M‐1	√				37.663	28.300	12.332	6.787	11.193
M‐2		√			36.464	27.259	9.860	6.307	10.719
M‐3			√		35.659	26.474	10.317	8.488	11.387
M‐4				√	35.630	25.227	9.910	6.189	8.953
M‐5	√	√			35.710	28.578	11.481	6.309	9.432
M‐6	√		√		31.524	24.961	11.332	7.017	8.955
M‐7	√			√	34.255	25.578	11.565	5.349	9.071
M‐8		√	√		30.185	26.688	9.422	5.826	9.584
M‐9			√	√	32.583	26.371	9.352	6.875	11.407
M‐10		√		√	32.308	25.282	9.168	8.621	9.218
M‐11	√		√	√	24.782	24.377	9.656	7.850	10.797
M‐12	√	√		√	29.282	24.782	8.481	5.455	8.719
M‐13		√	√	√	24.748	22.583	8.102	**5.124**	9.403
M‐14	√	√	√		26.152	22.308	8.106	6.022	8.771
M‐15	√	√	√	√	**24.336**	**17.640**	**8.019**	5.349	**7.645**

**FIGURE 10:**
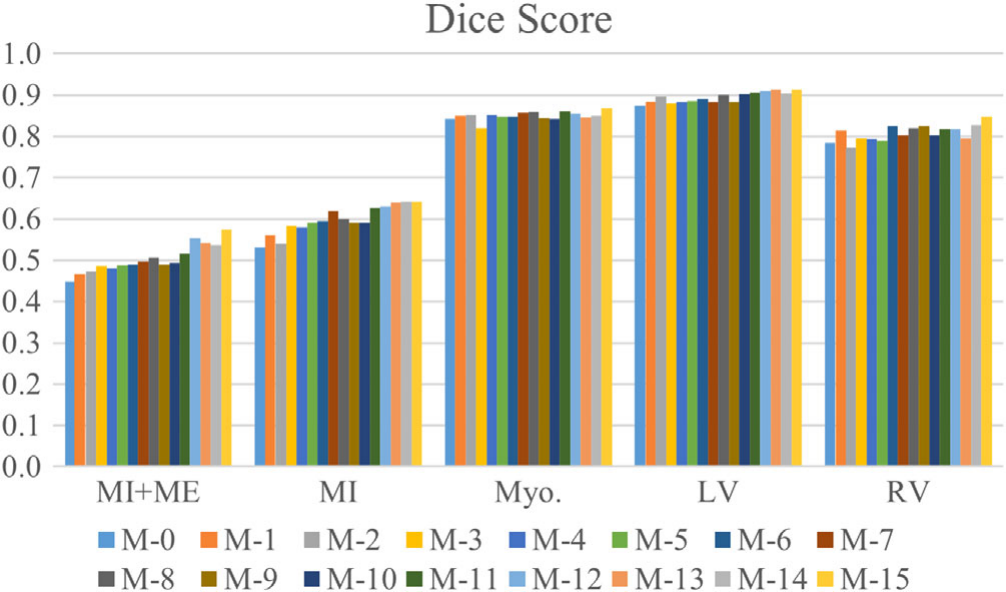
Histogram of dice segmentation effect of multi‐modal cardiac images under different ablation studies.

**FIGURE 11:**
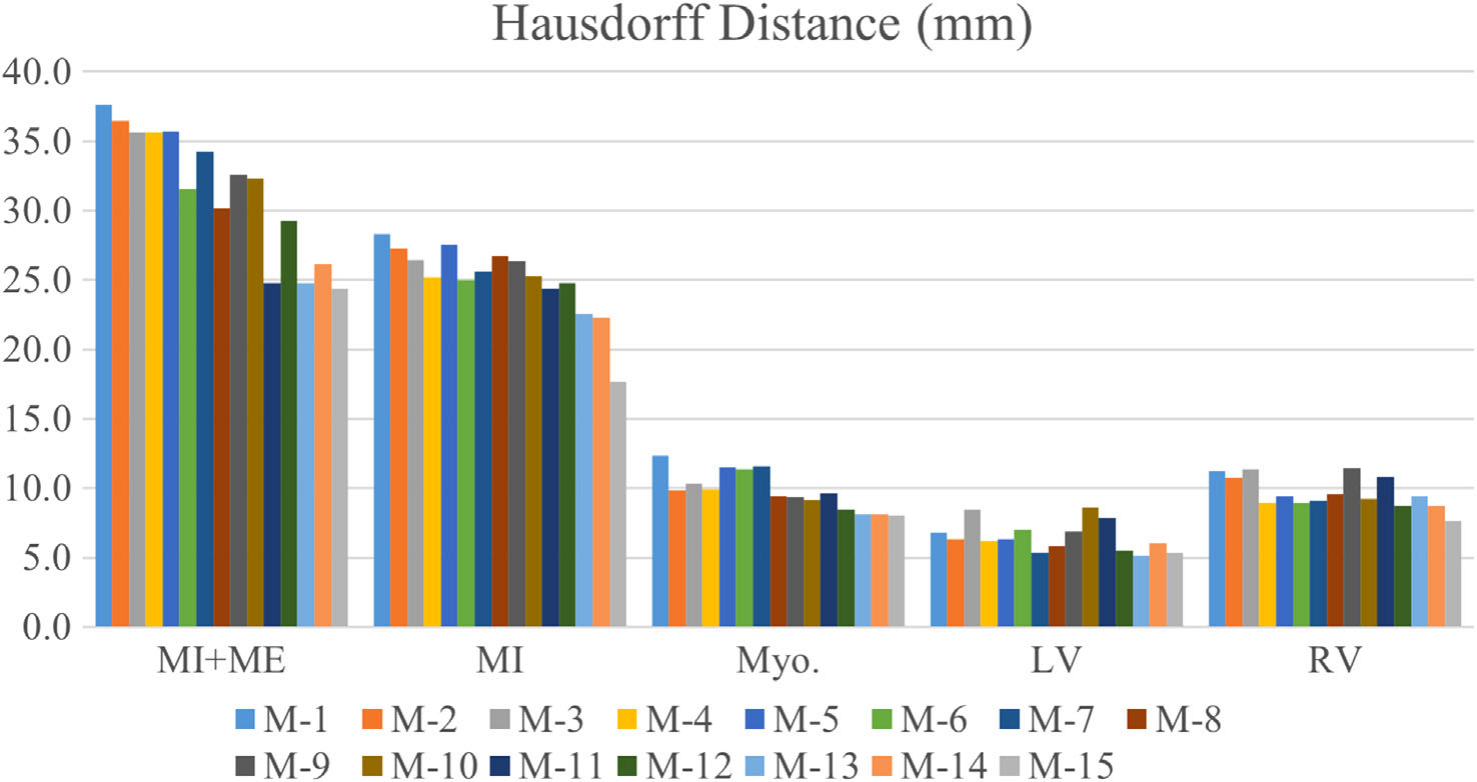
Histogram of HD segmentation effect of multi‐modal cardiac images under different ablation studies.

Figures 10 and 11 demonstrate the evaluation results of different ablation experiments on multi‐modal cardiac images. As can be seen from the figures, the NVTrans‐UNet presents better results, which shows the effectiveness of the NVT, MGF, and ASPP modules. It is worth noting that the gold standard label provided includes five parts, while myocardial infarction and edema are mostly considered.

## 5 DISCUSSION

In this paper, a multi‐modal cardiac image segmentation method NVTrans‐UNet is proposed. First of all, in the encoding phase, we introduce the NVT module. NVT is a hierarchical Transformer consisting of multiple neighborhood attention layers. The neighborhood attention adaptively locates the receptive field to the neighborhood around each token without the need for extra operations. And we introduce the local induction bias to reduce the computational cost. Moreover, we introduce the MGF module to distinct convolution layers of the encoder. The module aggregates the feature maps of task‐related information and automatically learns to adjust the contribution of the three modalities. Then, we introduce ASPP into the bottleneck layer. ASPP uses atrous convolution parallel sampling with different sampling rates, which can expand the receptive field to improve the segmentation capacity of small targets. Because the pathological regions of the cardiac image are usually relatively small and the boundary is fuzzy, the traditional segmentation loss effect is not ideal. We propose a mixed loss function that combines MS‐SSIM loss, Tversky loss, and Focal loss for segmentation at the pixel level and patch level, which can capture fine structures with clear boundaries and reduce the misclassification of the target region.

By redesigning the network structure and loss function, although NVTrans‐UNet shows better performance in multi‐modal cardiac image dataset, our model deals with 2D images and cannot fully utilize the 3D information of the data. We will make full use of the 3D information of the image in the future. Due to the nearby normal tissues will affect the segmentation precision of pathology regions. Subsequently, we will further explore the relationship between myocardial pathology and healthy tissues to enhance the segmentation precision of lesion regions. In addition, we will try to extend it to other small target segmentation tasks.

## 6 CONCLUSION

Automatic segmentation of cardiac images can assist doctors to diagnose cardiovascular diseases promptly, which has important clinical value. Therefore, we introduce a multi‐modal cardiac segmentation model NVTrans‐UNet based on deep learning to segment small target regions MI and ME, which provide a reliable diagnostic basis for physicians to make accurate judgments. With the advantage of NVT and MGF modules, NVTrans‐UNet can compensate for missing local information and fuse multi‐modal features dynamically which improves the accuracy of segmentation. On the MyoPS 2020 dataset, we obtained competitive results, the Dice score of MI was 0.642 ± 0.171 and the Dice score of MI + ME was 0.574 ± 0.110. The results of the experiments demonstrate the potential of our NVTrans‐UNet.

## AUTHOR CONTRIBUTIONS

Bingjie Li devised the project, performed the experiments, and drafted the manuscript. Tiejun Yang provided critical revision of the manuscript for important intellectual content, technical, and material support. Xiang Zhao contributed to the design of this study and the revision of the manuscript. All authors reviewed the results and approved the final of the manuscript.

## CONFLICT OF INTEREST

The authors declare they have no conflicts of interest.

## References

[1] Wang ZW , Hu SS . Interpretation of annual report on cardiovascular health and diseases in China. Cardiol Discov. 2019;1(4):269‐284. doi:10.1097/cd9.0000000000000040

[2] Scutti S . Nearly half of US adults have cardiovascular disease, study says. CNN. Published January 31, 2019. Accessed July 24, 2022. http://www.cnn.com/2019/01/31/health/heart‐disease‐statistics‐report/index.html

[3] Virani SS , Alonso A , Benjamin EJ , et al. Heart disease and stroke statistics—2020 update: a report from the American Heart Association. Circulation. 2020;141(9):e139‐e596. doi:10.1161/CIR.0000000000000757 31992061

[4] Yang X , Zhang Y , Lo B , et al. DBAN: adversarial network with multi‐scale features for cardiac MRI segmentation. IEEE J Biomed Health Inform. 2021;25(6):2018‐2028. doi:10.1109/jbhi.2020.3028463 33006934

[5] Vesal S , Gu M , Maier A , Ravikumar N . Spatio‐temporal multi‐task learning for cardiac MRI left ventricle quantification. IEEE J Biomed Health Inform. 2021;25(7):2698‐2709. doi:10.1109/jbhi.2020.3046449 33351771

[6] Tao X , Wei H , Xue W , et al. Segmentation of multimodal myocardial images using shape‐transfer GAN. Statistical Atlases and Computational Models of the Heart Multi‐Sequence CMR Segmentation, CRT‐EPiggy and LV Full Quantification Challenges. 2020:271‐279. doi:10.1007/978-3-030-39074-7_29

[7] Bi K , Tan Y , Cheng K , et al. Sequential shape similarity for active contour based left ventricle segmentation in cardiac cine MR image. Math Biosci Eng. 2021;19(2):1591‐1608. doi:10.3934/mbe.2022074 35135219

[8] Ammar A , Bouattane O , Youssfi M , Automatic spatio‐temporal deep learning‐based approach for cardiac cine MRI segmentation. Networking, Intelligent Systems and Security. 2021:59‐73. doi:10.1007/978-981-16-3637-0_5

[9] Cui H , Yuwen C , Jiang L , et al. Multiscale attention guided U‐Net architecture for cardiac segmentation in short‐axis MRI images. Comput Methods Programs Biomed. 2021;206:106142. doi:10.1016/j.cmpb.2021.106142 34004500

[10] Zhou T , Ruan S , Canu S , A review: deep learning for medical image segmentation using multi‐modality fusion. Array. 2019;3(4):100004. doi:10.1016/j.array.2019.100004

[11] Zhang Z , Liu C , Ding W , et al. Multi‐modality pathology segmentation framework: application to cardiac magnetic resonance images. Myocardial Pathology Segmentation Combining Multi‐Sequence Cardiac Magnetic Resonance Images. 2020:37‐48. doi:10.1007/978-3-030-65651-5_4

[12] Liao X , Qian Y , Chen Y , et al. Multi‐modality transfer learning network with adversarial training for 3D whole heart segmentation. Comput Med Imag Graph. 2020;85:101785. doi:10.1016/j.compmedimag.2020.101785 32898732

[13] Li W , Wang L , Li F , et al. Myocardial pathology segmentation of multi‐modal cardiac MR images with a simple but efficient Siamese U‐shaped network. Biomed Signal Process Control. 2022;71:103174. 10.1016/j.bspc.2021.103174

[14] Zhuang X . Multivariate mixture model for myocardial segmentation combining multi‐source images. IEEE Trans Pattern Anal Mach Intell. 2019;41(12):2933‐2946. doi:10.1109/tpami.2018.2869576 30207950

[15] Jiang H , Yang G , Huang K , et al. W‐net: one‐shot arbitrary‐style Chinese character generation with deep neural networks. Neural Information Processing. 2018:483‐493. doi:10.1007/978-3-030-04221-9_43

[16] Chartsias A , Papanastasiou G , Wang C , et al. Disentangle, align and fuse for multimodal and semi‐supervised Image segmentation. IEEE Trans Med Imag. 2021;40(3):781‐792. doi:10.1109/tmi.2020.3036584 PMC8011298 33156786

[17] He K , Zhang X , Ren S , et al. Spatial pyramid pooling in deep convolutional networks for visual recognition. IEEE Trans Pattern Anal Mach Intell. 2015;37(9):1904‐1916. doi:10.1109/tpami.2015.2389824 26353135

[18] Milletari F , Navab N , Ahmadi S‐A , V‐net: fully convolutional neural networks for volumetric medical image segmentation. 2016 Fourth International Conference on 3D Vision (3DV) . 2016. doi:10.1109/3dv.2016.79

[19] Wang Z , Simoncelli EP , Bovik AC , Multiscale structural similarity for image quality assessment. The Thrity‐Seventh Asilomar Conference on Signals, Systems & Computers, 2003. doi:10.1109/acssc.2003.1292216

[20] Abraham N , Khan NM , A novel focal tversky loss function with improved attention U‐Net for lesion segmentation. 2019 IEEE 16th International Symposium on Biomedical Imaging (ISBI2019). 2019. doi:10.1109/isbi.2019.8759329

[21] Lin T‐Y , Goyal P , Girshick R , et al. Focal loss for dense object detection. 2017 IEEE International Conference on Computer Vision (ICCV). 2017. doi:10.1109/iccv.2017.324 30040631

[22] Boykov Y , Kolmogorov V , Cremers D , et al. An integral solution to surface evolution pdes via geo‐cuts. Computer Vision – ECCV 2006. 2006:409‐422. doi:10.1007/11744078_32

[23] Salehi SS , Erdogmus D , Gholipour A . Tversky loss function for image segmentation using 3D fully convolutional deep networks. Mach Learn Med Imag. 2017:379‐387. doi:10.1007/978-3-319-67389-9_44

[24] Flett AS , Westwood MA , Davies LC , et al. The prognostic implications of cardiovascular magnetic resonance. Circulation. 2009;2(3):243‐250. doi:10.1161/circimaging.108.840975 19808599

[25] Beek AM , van Rossum AC . Cardiovascular magnetic resonance imaging in patients with acute myocardial infarction. Heart. 2010;96(3):237‐243. doi:10.1136/hrt.2009.172296 20133424

[26] Jiang H , Wang C , Chartsias A , et al. Max‐fusion U‐net for multi‐modal pathology segmentation with attention and dynamic resampling. Myocardial Pathology Segmentation Combining Multi‐Sequence Cardiac Magnetic Resonance Images. 2020:68‐81. doi:10.1007/978-3-030-65651-5_7

[27] Jegou S , Drozdzal M , Vazquez D , et al. The one hundred layers tiramisu: fully convolutional DenseNets for semantic segmentation. 2017 IEEE Conference on Computer Vision and Pattern Recognition Workshops (CVPRW). 2017. doi:10.1109/cvprw.2017.156

[28] Zhang X , Noga M , Punithakumar K , Fully automated deep learning based segmentation of normal, infarcted and edema regions from multiple cardiac MRI sequences. Myocardial Pathology Segmentation Combining Multi‐Sequence Cardiac Magnetic Resonance Images. 2020:82‐91. doi:10.1007/978-3-030-65651-5_8

[29] Ronneberger O , Fischer P , Brox T . U‐Net: convolutional networks for biomedical image segmentation. Lecture Notes in Computer Science. 2015:234‐241. doi:10.1007/978-3-319-24574-4_28

[30] Zhai S , Gu R , Lei W , et al. Myocardial edema and scar segmentation using a coarse‐to‐fine framework with weighted ensemble. Myocardial Pathology Segmentation Combining Multi‐Sequence Cardiac Magnetic Resonance Images. 2020:49‐59. doi:10.1007/978-3-030-65651-5_5

[31] Ma J , Cascaded framework with complementary CMR information for myocardial pathology segmentation. Myocardial Pathology Segmentation Combining Multi‐Sequence Cardiac Magnetic Resonance Images. 2020:159‐166. doi:10.1007/978-3-030-65651-5_15

